# Investigating the conditions in which women GPs thrive in general practice: a realist review

**DOI:** 10.3399/BJGP.2025.0785

**Published:** 2026-08-11

**Authors:** Ruth Abrams, Vickie Williams, Sophie Park, Su Golder, Aaliya Goyal, Lilith Whiley, Laura Jefferson

**Affiliations:** 1 University of Surrey, Guildford, UK; 2 Nuffield Department of Primary Care Health Sciences, University of Oxford, Oxford, UK; 3 Department of Health Sciences, University of York, York, UK; 4 independent, NHS GP, London, UK; 5 Sussex Business School, Brighton, UK; 6 School of Health Sciences, University of Manchester, Manchester, UK

**Keywords:** general practice, general practitioners, practice organisation, women GPs, workforce

## Abstract

**Background:**

Women GPs experience higher rates of burnout, anxiety, depression, and slower career progression than men GPs. Women GPs are sought out by patients for specific conditions, which may increase consultation length. This can be a source of job satisfaction for some. However, it can also contribute to additional exhaustion and affects career progression, meaning women GPs are at higher risk of leaving the profession. There is an urgent need to identify solutions to retain and support them.

**Aim:**

This review brings together a wide range of evidence to identify the conditions in which women GPs can thrive, to better support them at work.

**Design and setting:**

An international realist review of academic and grey literature was conducted across eight databases and Google Scholar.

**Method:**

Following a realist approach, supported by stakeholder and patient involvement, a range of published documents were analysed using a realist logic to identify how, why, for whom, and in what circumstances women GPs thrive at work.

**Results:**

A total of 72 documents are included in this review. Sixteen configurations describe the conditions in which women GPs can thrive. Results cover the following three concepts: patient-facing work; navigating competing roles; and sustainable careers, leadership, and success. However, literature also places heavy reliance on encouraging women GPs to ‘fix’ their own circumstances.

**Conclusion:**

This review provides evidence-based recommendations that may be of interest to those responsible for organisational culture within general practices, including (but not limited to) GP partners, and women GPs, who can utilise the recommendations to influence change.

## How this fits in

To date, there is limited research into the general practice workforce and how specific groups can be supported to thrive. Women GPs, in particular, are at higher risk of burnout and experience more career challenges. They also have a higher intention to leave the profession than men. This review shifts from a narrative of describing workforce challenges, to identifying the available evidence into how women GPs can thrive, with a view to supporting and retaining them. Greater focus now needs to address the following two major gaps: 1) the evaluation of initiatives capable of supporting women GPs; and 2) moving beyond individualised solutions.

## Introduction

Strategic health policy goals, including the NHS 10 Year Plan for England, aim to shift care from hospitals into community settings.^1^ Yet rising patient demand and reduced workforce capacity have led to a workforce crisis in primary care that limits this potential.^2,3^ In 2021, GP appointments increased by 370 million consultations, up 18.5% from 2019.^4^ However, many GPs are reducing clinical hours, and one-third say they plan to leave medical practice within the next 5 years.^5^ While the wellbeing and retention of GPs has gathered increasing research and policy focus over recent years, evidence tends to focus on describing the challenges. There is an urgent need to identify successful workforce solutions to support GPs to thrive.

To thrive at work is to experience vitality, learning, and a positive psychological state.^6^ Thriving is a well-established psychological concept associated with both individual characteristics (for example, proactive personality, emotional stability, and core self-evaluation) and workplace factors (for example, job demands and resources, training opportunities, flexible working, recognition, mentoring, social support, co-worker behaviour, organisational culture, and leadership behaviour).^6–8^ Thriving at work has been connected to several critical staff outcomes, with employees reporting less burnout, increased job performance, and higher rates of job satisfaction and organisational commitment.^7,9^

The concept of thriving at work is pertinent to all GPs, but especially so for women GPs. Existing evidence shows that women GPs are affected more by lower rates of career progression, and higher rates of stress, emotional exhaustion, and anxiety at work.^10–12^ Women GPs also experience one of the worst pay gaps in medicine despite them making up more than half of the GP workforce.^12^ Patients seek them out for specific conditions, and value being seen by them.^13–16^ Yet these conditions often require them to spend longer with patients, more frequently discussing patients’ feelings and emotions.^3,13–16^ Thus, they represent a key demographic to support and retain. Indeed, the Royal College of General Practitioners contends that retaining this staff group is one of five priorities for future policy and research.^4^

To date only one review exploring the working conditions of women GPs has been undertaken, by Jefferson *et al*,^17^ with a focus on barriers to career progression. Findings identified that no specific interventions exist to support women GPs to progress in their careers, and that societal expectations, workplace cultures, and partner roles all shape whether and how a woman GP can progress at work.^17^

To progress this field of research, the review of evidence presented in this paper identifies a broader range of conditions in which women GPs can thrive, considering outcomes including individual wellbeing, job satisfaction, career progression, and organisational performance.

## Method

This review was informed by the following research questions:

1. What are the mechanisms acting at an individual, team, organisational, and societal level that affect women GPs’ health and wellbeing, performance, and carer progression?
2. In what contexts are these mechanisms triggered (or not triggered)?
3. What outcomes (both intended and unintended) do these contexts and mechanisms lead to?
4. What interventions or strategies can be implemented to help women GPs to thrive at work, based on relevant contexts, mechanisms, and outcomes?
5. What are the critical gaps in the literature (including the views and experiences of women and minoritised women)?

This review takes a realist approach to identifying the conditions in which women GPs can thrive at work.^18^ Evidence was sought that was capable of describing how women GPs experience their work, allowing for a number of interacting contexts, mechanisms, and outcomes to be determined through a realist logic of analysis. A realist approach seeks to map these interactions in the form of generative causation, leading to an evidence base that can indicate what works, for whom, how, and under what circumstances. Findings are then synthesised into organising constructs (for example, concepts) that integrate multiple context–mechanism–outcome configurations (CMOCs), which bring together causal explanations across contexts.

Box 1 (Supplementary Information S1-S6) outlines how this approach was applied, in line with a realist approach,^18^ and reports on these in line with RAMESES guidelines.^19^ The protocol for this work is also available.^20^

**Box 1.** Overview of applied realist approach

Step 1: locating existing theories	Existing theories were initially located in MEDLINE using the search strategy built on previous work undertaken by Jefferson *et al* ^17^ and tailored to the Goh *et al* ^7^ conceptual model of thriving. This initial search informed the initial programme theory, alongside project team, PPI, and stakeholder input (Supplementary Information S1)
Step 2: searching for evidence	The primary, exhaustive search conducted in November 2024 and updated in October 2025 aimed to identify empirical evidence of the range of conditions likely to influence women GPs’ ability to thrive in general practice (Supplementary Information S2a). The search strategy was developed and conducted by an information specialist (the fourth author). Databases searched were MEDLINE, Embase, CINAHL Ultimate, PsycInfo, Science Citation Index (SCI), Social Science Citation Index (SSCI), and Scopus. In addition, a search of Google Scholar was undertaken
Screening	Searches identified 844 results. The first and second authors screened 10% for consistency at title and abstract stage. Remaining documents were screened by the second author in consultation with the first author. In total, 157 documents were included for full-text screening, of which 10% were screened by the first author. Of the 157 full texts screened, 57 documents were identified for analysis by the second author. Of these 57, the first author screened a further 10%. Google searches and citation tracking also took place during December 2024. While 28 documents were located, only one document was included (Figure 1)
Additional searching	An additional targeted search was undertaken in July 2025, to capture specific interventions using targeted terms identified in papers from the exhaustive, primary search including: mentoring and coaching (see Supplementary Information S2b for details of this search strategy). In total, 170 results were located. The first and second authors screened 10% of the document’s titles and abstracts for consistency. The remaining documents were then screened by the second author. Thirty documents were included for full-text screening, with a 10% random sample reviewed by the first author. Fourteen documents were included for analysis. (Figure 2)
Step 3: document selection	A total of 72 full-text documents were selected for inclusion based on their data relevancy to the review. Inclusion and exclusion criteria: this included all documents from 2012 onwards, addressing women GPs working in general practice or primary care, within all OECD countries (except countries with different primary care model to the UK), as well as documents capable of identifying a gendered focus to strategies or interventions that support women GPs at work to facilitate outcomes of interest: health and wellbeing, career development and performance
Step 4: data extraction	All included documents were coded using NVivo (version 15) (see Supplementary Information S3 for document characteristics and Supplementary Information S4 for details of the coding framework)
Step 5: data synthesis	Working across and within coded data extracts, context–mechanism–outcome configurations (CMOCs) were developed as part of an iterative development of causal explanations (see Supplementary Information S5 for illustrative data reflective of each CMOC)
Step 6: refine programme theory	The final programme theory (Figure 3) was refined through discussion with the GP stakeholder and PPI group

OECD = Organisation for Economic Co-operation and Development. PPI = patient and public involvement.

### Searches

The search strategy for this study modified an existing search developed by the fourth and seventh authors for their study into career barriers for women GPs.^17^ Modifications included the introduction of core concepts found in thriving literature including but not limited to the following: job satisfaction; wellbeing; and performance.^7^

**Figure 1.**
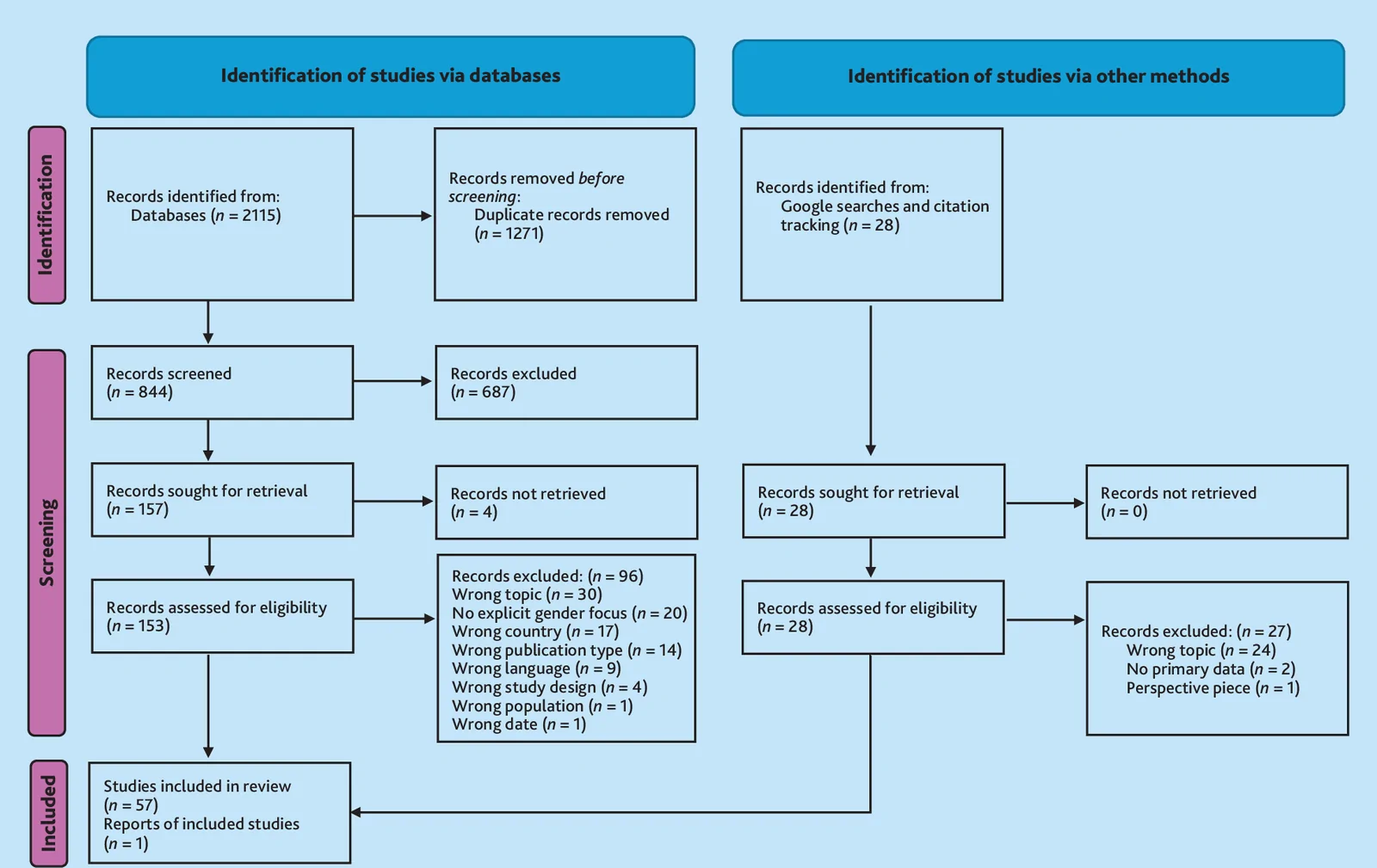
Primary searches.

**Figure 2.**
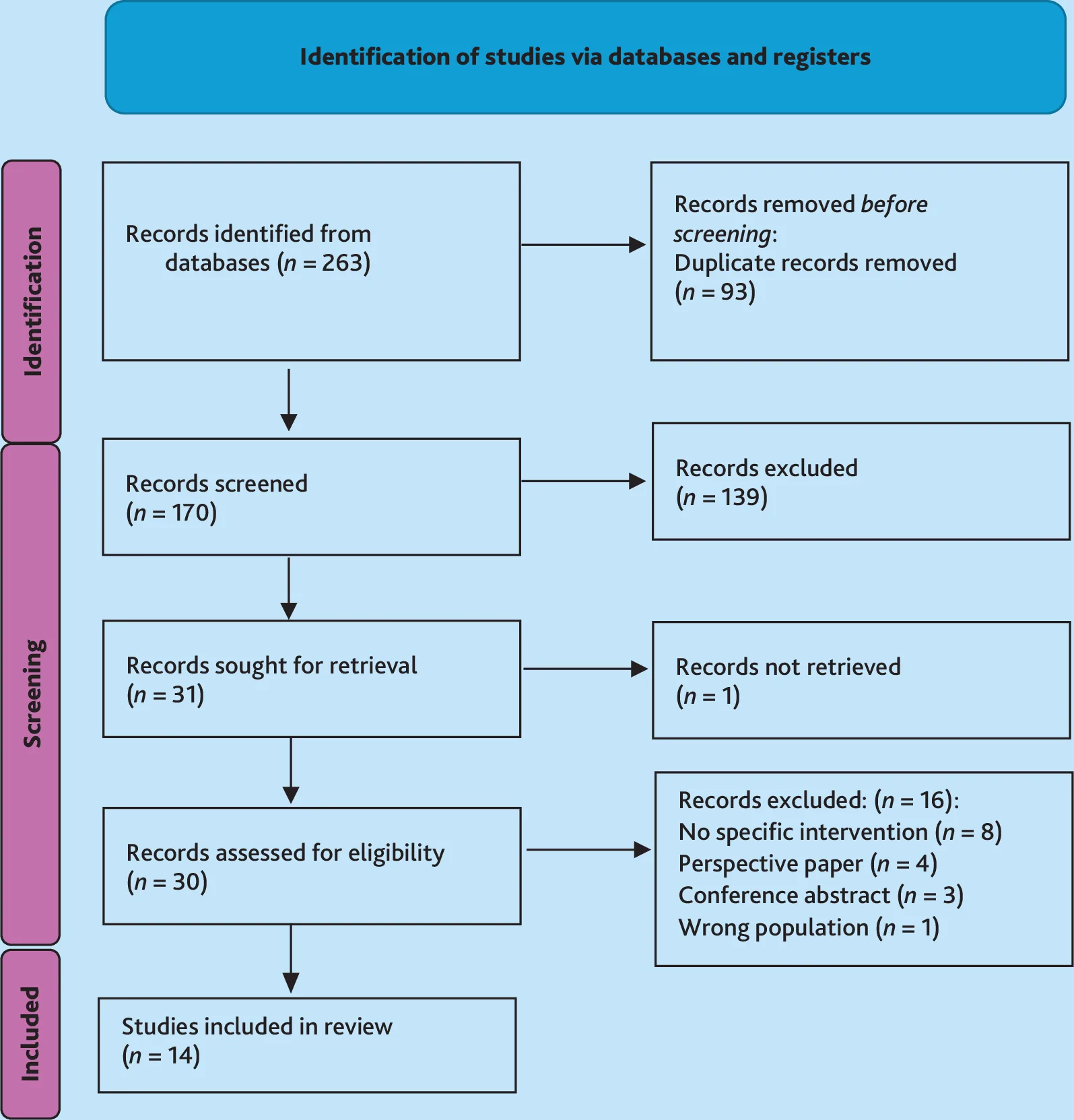
Additional searches.

**Figure 3.**
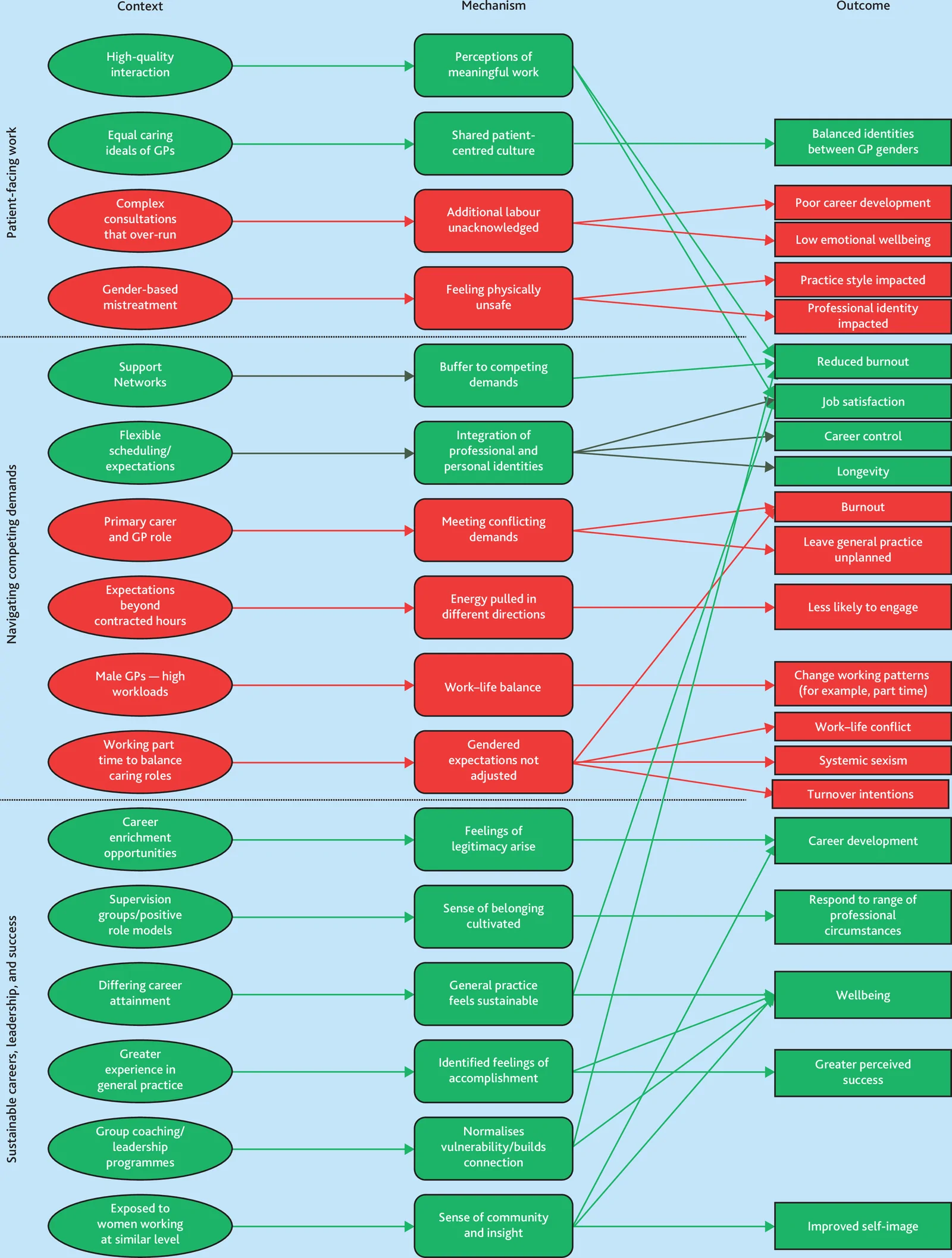
Final programme theory.

GP stakeholders and patient and public involvement (PPI) members were involved throughout the research, helping to establish the initial programme theory and refine the final programme theory (see Supplementary Information S6 for details).

## Results

A total of 72 documents are included in this review, spanning primary healthcare systems in: European countries (*n* = 25); UK(*n* = 18); US (*n* = 13); Australia (*n* = 8); Canada (*n* = 5); multiple countries (*n* = 2); and Mexico (*n* = 1). Academic and empirical literature comprised 96% (*n* = 69) of total documents included. Using a realist logic of analysis, a total of 16 CMOCs are mapped across three concepts including: 1) patient-facing work; 2) navigating competing roles; and 3) sustainable careers, leadership, and success. In the following narrative not only are findings presented that show how women GPs may thrive, but also the conditions that undermine their ability to do so consistently.

### Patient-facing work

Women GPs value a patient-centred approach to care and spend longer in consultations, asking psychosocial questions, exploring patient concerns and expectations, as well as evaluating and explaining treatment choices.^21–23^ This holistic dimension of being a GP stimulates greater patient disclosure, information, and referrals, while limiting unnecessary prescriptions.^21,22,24^ These are aspects of the role that lead to a sense of job satisfaction, because women GPs find it meaningful^21,25^ (CMOC 1). However, women GPs can also attract more psychosocially complex patients (for example, those with multiple and intersectional diagnoses, mental health and women’s health appointments) because they are assumed and expected to demonstrate greater empathy, attentiveness, and thoroughness. This can result in increased time spent with individuals at the expense of reduced consultations.^26–31^ These gendered attitudes are costly to women GPs, leading to longer working hours to fulfil patient demands and expectations. This has implications for earnings capacity (in volume-based remuneration systems), and exacerbates their risk of burnout^26–29^ (CMOC 2).

Women GPs are also more likely to experience sexual harassment and discrimination, from both patients and colleagues, than men GPs.^32^ Gender-based mistreatment ranges from general sexist remarks or behaviours, such as doubting their clinical ability or assuming they are more junior or a different professional because of their gender, to inappropriate sexual or coercive advances, and may negatively impact women GPs’ style of practice.^33^ These encounters are intensified for women GPs with intersectional identities (such as global majority and/or disabled).^34,35^ These gender disparities may threaten women GPs’ professional identity, self-esteem, and career development,^32,36^ and increase the risk of isolation, dissatisfaction, and burnout,^26^ if women GPs feel physically and psychologically unsafe at work (CMOC 3).

When a patient-centred approach to care is upheld by all GPs, it may help to shift the unequal distribution of longer consultations that are shouldered by some women GPs.^29^ Integrating practical insights into how communication strategies and empathy shape clinical care, and targeting universal interventions for medical students and GPs aimed at enhancing patient outcomes, can support the cultivation of a patient-centred ethos^37^ (CMOC 4) (Box 2).

Box 2. **CMOCs 1–4**

CMOC 1: When women GPs encounter high-quality, enriching interaction with patients and colleagues (C), then they are more likely to have job satisfaction and less likely to burn out (O), because this feeds their perceptions of meaningful work (M)
CMOC 2: However, when patients with complex health issues request to see women GPs and these appointments take longer (C), then women GPs’ career development and emotional wellbeing is negatively impacted (O), because they operate in a system that does not acknowledge this additional labour (M)
CMOC 3: When women GPs experience gender-based mistreatment from colleagues and patients (C), then this may negatively impact their style of practice, professional identity, and career development (O), because some women may feel psychologically and/or physically unsafe at work (M)
CMOC 4: However, when all GPs are upheld to the same caring ideals (C), then professional identities and expectations become better balanced between genders (O), because collaborative and empathetic patient-centred cultures are shared (M)

### Navigating competing roles

Social support networks are essential in providing flexibility for women GPs who balance competing caring roles.^25,38–41^ Social support networks can help to buffer workplace stress and burnout, enhance opportunities for career success, and support thriving within general practice^41^ (CMOC 5). However, women GPs balancing dependent care remain at higher risk of chronic stress, burnout, and leaving general practice unplanned,^25,42,43^ because they are still expected, by society and themselves, to excel simultaneously in multiple roles (for example, mother, partner, and doctor).^38,44^ This gendered division of labour can be more pronounced for women GPs of racial and ethnic minorities owing to cultural expectations that construct women’s primary role as within the home^27,38^ (CMOC 6). It also affects younger GPs with young children indicating significant barriers for early career practitioners with caring responsibilities.^30,41,43,44^

Expectations to work beyond contracted hours restricts the involvement and development of women GPs who are also primary caregivers.^40^ Their energy (ranging from transient tiredness to burnout) is already overextended in balancing clinical duties with raising children, which limits their availability for after-hours meetings, care, and research.^45^ This may delay women GPs’ taking on additional roles in leadership, education, supervision, and training.^46^ In settings where GPs can buy a share in the practice ownership, this may delay their decision, often waiting or re-engaging with these activities when their children have grown up.^26–28,46^ This can impact career development and success.^27,35,40,41^ Expectations to work overtime and the lack of control over scheduling, simultaneously increases women GPs’ career dissatisfaction and burnout, while fuelling their decision to work part-time^26,32^ (CMOC 7).

Flexibility in scheduling and expectations can support women GPs to maintain work–family balance.^26,30,35^ Women GPs are also increasingly diversifying their career options and engaging in job crafting to garner greater temporal autonomy (for example, sessional or salaried posts, portfolio careers).^41,47^ Navigating work–family life in this way supports women GPs’ sense of career control, satisfaction, and longevity in general practice^30,48^ (CMOC 8). Working part-time is an increasingly common trend in general practice.^47,49,50^ (Part-time work in UK general practice is defined as a GP working less than eight clinical sessions per week. A clinical session defined by the British Medical Association [BMA] is four hours and 10 minutes. However, GPs working part-time can often work or exceed full-time hours owing to the intensity and complexity of consultations, administrative duties, and additional role responsibilities. For example, a six-session week can amount to 36 hours or more, therefore part-time work can be an inaccurate reflection in practice.)^51^ This trend includes men GPs who are reducing practice hours to mitigate demanding workloads^41,49,50^ or achieve a better work–life balance^49,52^ (CMOC 9). However, for women GPs, who adopt part-time work to mitigate work–life conflict, they may still experience significant pressures because part-time work is not acknowledged or valued within the profession.^29,53^ They experience differential treatment and respect from colleagues and patients by working part-time,^27,29^ which again contributes to burnout and turnover intentions^27,29,43,53^ (CMOC 10) (Box 3).

Box 3. **CMOCs 5–10**

CMOC 5: When women GPs with additional caring responsibilities have access to a network for psychological and/or practical support from family, friends, and childcare (C), then it reduces their likelihood of burnout (O), because it provides a buffer to competing demands (M)
CMOC 6: However, when women GPs are responsible for dependent care (for example, child-rearing, looking after older parents) in addition to their role as GP (C), then they are more likely to experience chronic stress, burnout, and leave general practice unplanned (O), because they are trying to meet conflicting demands (M)
CMOC 7: However, when role expectations or career development opportunities include work outside of contracted hours and women GPs have existing care responsibilities (C), then they may be less likely or able to engage with these opportunities (O), because their energy is already pulled in too many different directions (M)
CMOC 8: When there is flexibility in appointment scheduling and role expectations for women GPs with dual caring roles (C), then their sense of career control, satisfaction, and longevity may be improved (O), because they can better integrate professional and personal identities (M)
CMOC 9: When men GPs experience high workload and work-related issues (C), then they are also likely to change their working patterns (for example, working part-time or retiring early) (O), because they too want a better work–life balance (M)
CMOC 10: When women GPs work part-time to fulfil additional roles (C), then they may still experience high workloads, work–life conflict, burnout, systemic sexism, and turnover intentions (O), because gendered expectations are not adjusted for part-time working (M)

### Sustainable careers, leadership, and success

The proactive provision of career enrichment opportunities and support is a valuable and effective strategy that nurtures women GPs for a successful and sustainable career in general practice.^28,30,35,38,40^ Women GPs can source relevant and timely support in response to challenges at different career and life stages, harnessing skills and education to progress their career at the right time^38,40^ (CMOC 11). Access to supervision groups, being mentored by positive role models, and having harmonious practice dynamics, facilitates a sense of safety and belonging for women GPs.^27,54^ This is because women GPs value opportunities to better understand and manage the complexity of patients who seek their time while navigating challenging working conditions^54^ (CMOC 12).

Perceptions of success and satisfaction may differ among women GPs.^46,48^ For example, medical mentoring may be more appealing than professional leadership positions.^45^ Job crafting can support women GPs to rediscover optimism in their role and career trajectory as this fosters agency to balance multiple roles with meaningful aspects of their job.^29,42,53^ Allowing for differing perceptions of career attainment may increase feelings of wellbeing and job satisfaction because it drives sustainable careers in general practice^29^ (CMOC 13). Women GPs with greater experience in primary care report greater wellbeing and perceived success because they have developed strategies to respond to gender-specific challenges^28,29,55^ (CMOC 14).

Potential initiatives that may support these aims include the following: virtual and group coaching programmes; closed interpersonal online social groups; implicit biases training; ‘doctor’ badges; gender-specific conferences, consortiums, leadership training summits and professional development and leadership programmes.^56–67^ These initiatives, while not yet evaluated, should be accessible to all women, particularly at the outset of their careers.^56,63,64,66^ Group-based components, including coaching, and the ability to be around other women, were particularly beneficial because they allowed women to feel a sense of community.^57,59,63,67^ These initiatives often led to improved feelings of wellbeing, and career development opportunities including identifying mentors and sponsors^56,58–60,62,64^ (CMOC 15–16) (Box 4).

Box 4. **CMOCs 11–16**

CMOC 11: When women GPs are given opportunities for career enrichment (mentorship, role models, leadership education and skills) (C), then they may feel more equipped for career development (O), because feelings of legitimacy arise (M)
CMOC 12: When women GPs can access and make use of supervision groups and see positive role models in practice (C), then they may be more able to respond to a range of different professional circumstances (O), because a sense of belonging and safety has been cultivated (M)
CMOC 13: When women GPs are given opportunities for differing perceptions of career attainment (C), then they may be more likely to experience positive perceptions of wellbeing and job satisfaction (O), because they have made general practice feel sustainable (M)
CMOC 14: When women GPs have worked in general practice for longer (C), then they are more likely to experience greater wellbeing and perceived success (O), because they have identified what makes them feel accomplished (M)
CMOC 15: When coaching or leadership programmes contain a group component (C), then women physicians may experience a decrease in distress and burnout and improvement in wellbeing (O), because it normalises vulnerability and builds a culture of connection (M)
CMOC 16: When women are exposed to other women working at a similar level to them, in a learning environment (C), then they may experience improved self-image and career development (O), because they have benefitted from a sense of community and insight (M)

## Discussion

### Summary

This review broadens the current understandings of both working conditions, and the mechanisms through which thriving may occur by using the literature as the object of this analysis, rather than the authors’ viewpoint or belief system.^68^ In analysing literature in this way, there appear to be multiple conditions that, when present, may support women GPs to thrive. These include: high-quality interactions between patients and colleagues, which see all GPs as equal; access to social support networks; flexible appointment scheduling; career enrichment opportunities; access to supervision groups and positive role models; and visibility of different career models. However, these run parallel to conditions that equally undermine a woman GP’s ability to thrive, including gender-based mistreatment; unequally distributed patient lists and requests; career juggling; conflicting role expectations; and career development opportunities outside of contracted hours.

In viewing literature as the object of the analysis, dominant narratives were identified that mean the evidence base focuses predominantly on the ‘choices’ made by women GPs to fix their own situations. This includes an emphasis on part-time working, or job crafting, which has a subsequent impact on patient access and care. Rarely are organisational drivers of these working conditions challenged.^26,47^ There is also an assumption in the literature that most women perform dual caring responsibilities. However, women are not a homogeneous group and there are women for whom dual caring responsibilities do not apply. Finally, it is recognised that some of the issues raised in this review also affect men, or are issues that can only be addressed if men are also supported. As the GP workforce grows more diverse, research is now needed on how different identities shape these experiences, to build a stronger evidence base.

In conclusion, this review has identified 72 documents, analysed using a realist approach, to show the conditions in which women GPs can thrive at work. Women GPs find high-quality interactions with patients enriching and meaningful; at the same time, this can lead to burnout. Juggling multiple caring roles outside of work, gender-based mistreatment at work, and a system that does not always reward equally, makes general practice work especially challenging for women GPs, limiting their ability to thrive. Crucially, these findings point to the ways in which women GPs can be supported to thrive, conditions that now need implementing among colleagues, patients, the NHS system, and regulatory bodies.

### Strengths and limitations

This paper situates women GPs’ experiences within wider societal and gendered expectations of care, showing how professional identities intersect with both emotional labour and caring identities. These multiple and often competing identities can leave women GPs carrying disproportionate personal and professional burdens. Indeed, those working in general practice with childcare responsibilities have been shown to have challenges finding childcare to accommodate their work schedules.^69^ By showing these burdens through the articulation of these findings, this review challenges the implicit assumption that clinicians, particularly women, can and should divorce caring responsibilities from professional identity.

Methodologically, a realist review offers transferability of these findings to other specialisations across the healthcare sector (for example, cardiology, endocrinology, oncology, and so on), as well as different employee groups (for example, nurses, healthcare assistants) and also potentially those in non-clinical roles. These findings may also apply to countries experiencing workforce crises including both Canada and Australia, who have similar workforce retention issues. By looking through the lens of generative causation, evidence can be holistically considered by drawing it together through patterns of causation across different contexts. This does, however, also indicate a limitation in that the process of bringing configurations together requires interpretive judgement.^18^ This introduces an element of subjectivity that the authors have sought to address through regular team meetings, active stakeholder engagement, public engagement, and appropriate reporting.^19^ These findings were also guided by an evidence-based conceptual model of thriving.^7^ This necessarily placed boundaries around this review, but in doing so may have excluded other relevant areas such as women GPs’ experiences of complaints processes, regulatory scrutiny, and professional accountability.

This review also highlights critical gaps in evidence. First, there are racialised and ethnic structural barriers that are considerably under-explored in the current evidence base. While this review shows that, across some areas, women GPs from ethnic minority backgrounds disproportionately experience discrimination, there is now a need for greater intersectional research. Future intersectional research needs to also focus on experiences of women GPs who are neurodivergent, disabled, or living with long-term health conditions, which remain entirely absent from the literature. Second, the authors identified only one document^35^ that highlights systemic changes required to support women GPs’ reproductive health at work, and only one existing review bringing together evidence on women GPs’ career progression.^17^ Given that women GPs are likely to experience menstruation, pregnancy, postnatal return to work, and/or menopause, this is a considerable omission in the evidence base,^70,71^ and particularly so in regard to how this might interact with their career progression and sustainability. Finally, there is also a dearth of performance-related data at an organisational level in regard to thriving, which indicates this is an area in need of focus.

### Comparison with existing literature

Existing evidence shows that pathways to sustaining workplace thriving include the following: 1) engaging in self-care; 2) the creation and maintenance of high-quality relationships; and 3) having a community both inside and outside work.^9^ Applying the conceptual lens of thriving at work from the psychological literature^7,9^ has enabled the identification of indicative outcomes to show how women GPs can thrive at work. Many of the mechanisms triggered in the authors’ configurations share similarities with the antecedents of thriving models, including the following: perceptions of meaningful work; psychological safety; energy management tactics; professional identity; role conflict; work–life balance; role expectations; belonging; accomplishment; connection; community; and heedful relating.^7,9^

Job crafting and energy management were both evident in the review and relate to existing literature framed as ‘self-care’ in the thriving literature.^9^ High-quality relationships (among both patients and colleagues) do encourage workplace thriving, particularly when they reflect respect, civility, and the ability to help others.^72^ Naturally, when incivility is present, the ability to thrive is reduced.^9^ Having a professional community within work environments, and a supportive community outside of work were evidenced to support women GPs thriving in this review. Thriving literature indicates that these work best when they are varied, including, for example, activity-based groups, communities of practice, online communities, local neighbourhood communities, and the organisation’s community. The recommendations outlined in Box 5 evidence how these can be implemented for women GPs.

**Box 5.** Recommendations for practice

Level	Recommendation
Interpersonal	Establish a shared understanding that women GPs often handle complex, emotionally demanding appointments and adapt systems in equitable ways that reflect and support this (CMOC 2) Support efforts to acknowledge and redistribute this workload fairly across the team where possible (CMOC 4) Consider if meetings and extra-curricular activities are organised in parenting and carer-friendly hours (CMOC 7–8) Discuss and respect colleagues’ need for flexible working arrangements and part-time working, especially those with dual caring roles (CMOC 9–10) Reflect and mitigate for individual and team-based dynamics, assumptions, and interactions: do they reinforce or challenge bias? (CMOC 3–4)
Organisational	Protect time for high-quality and enriching interactions with colleagues and patients by scheduling time for peer-to-peer connections and longer consultations (CMOC 1) Cultivate a working environment where it feels safe to report or speak up when witnessing gendered comments or unfair treatment. Reporting policies need to be visible and accessible to encourage others to use them safely, including patients (CMOC 3) Implement and evaluate initiatives that signal how staff can challenge implicit biases (CMOC 3) Develop and evaluate family-friendly policies to support all GPs in balancing multiple roles (CMOC 5–8) Review how tasks and appointments are allocated and consider whether they are proportionate to contracted hours (CMOC 10) Include part-time GPs equitably in career development opportunities and decision making (CMOC 10, 11, 13) Consider data capture and reporting on metrics relating to gender and thriving across practices in regard to CMOC 1–16
Regulatory bodies; training providers	Implement and evaluate initiatives that actively foster and encourage the following: Participation in networks and forums including gender-specific conferences, training summits, and professional development programmes (CMOC 16) Opportunities to learn from women GPs with greater experience of working in general practice (CMOC 14) Participation in supervision groups, group coaching, and leadership programmes (CMOC 11, 12, 15) Opportunities for early career GPs to learn the supervisor role by shadowing more experienced and senior supervisors (CMOC 12) Offer formalised support from a named mentor (men or women) (CMOC 11) Celebrate and make visible diverse career paths in general practice and encourage positive role models in practice (CMOC 13)
Broader society including patient groups and the general public	Reflect on the expectations held about GPs including the associations and assumptions when the word doctor or GP is used (CMOC 3) Celebrate the diversity of ways in which general practice can be done and ensure systems and reward mechanisms reflect this. Where mismatches are identified between expectations and experience (for example, different meanings attributed to the word doctor or GP in different contexts), highlight the need for diversity and equity across this range of roles and expertise Develop awareness of any unconscious (or conscious) biases or assumptions about how women GPs are expected to perform their role (CMOC 2–4)

Thriving literature does, however, place an emphasis on personal agency, without always considering wider, organisational and societal responsibilities for workplace thriving. The evidence base shows how, in the current system, women GPs are largely held responsible for their own thriving. This finding shares similarities with the work of Jefferson *et al.* ^17^ For example, in their review into women GPs and barriers to career progression, findings show that women GPs bear the brunt of caring responsibilities, shaping their ability to opt for partner roles or not. This is especially in the absence of training models, work systems, and career structures that are inclusive and integrate the different life experiences women navigate and negotiate.^73^ Findings also indicate that flexibility in hours and discriminatory workplace practices also shape career progression, and that social support, flexible working, role models, and leadership development may all support women GPs.^17^

The authors seek to draw awareness to this within the broader context of work in general, which includes aspects of neoliberalism and its related demands.^74^ This is particularly heightened in the care sector because of socialised gender norms of women and of doctors.^75^ Therefore, while this review points to the conditions in which women GPs can thrive, and indeed are thriving, this remains a limited lens if not complemented by wider systemic changes (for example, organisational structures, policies, and organisational practices).

### Implications for research and practice

The evidence indicates multiple ways women GPs thrive at work, achieved through the illustrative outcomes of job satisfaction, wellbeing, career development, and professional identity. How this research can be translated across research, policy, and practice to aid the translational value of the findings is described below.

Regarding research, throughout this paper several areas for future investigation have been indicated. These include: a greater emphasis on intersectional research, with a specific focus on how women GPs from racial and ethnic minorities experience work, and also the experience of women GPs who are disabled, living with long-term health conditions, or are neurodivergent.

Given the considerable attention within existing literature on women GPs’ experiences of navigating the profession alongside caring for children, future research is still needed into evidence-based solutions to overcome systemic barriers to juggling a career and childcare. Research is also needed to understand the experiences of women GPs who are childless and childfree, and what initiatives support them to thrive.

Another area of research is around the implementation and evaluation of initiatives capable of supporting women GPs including: gender-specific conferences, training summits and professional development programmes; group coaching, supervision, and leadership programmes; formalised mentorship; and visual identification aids. However, these current initiatives may reinforce individualised solutions, where caution must be urged.

Supplementary Information S7 also shows statements that, while of interest to this study, could only be partially supported by the data and therefore warrant further testing through empirical data collection. These include the following: how the encouragement of non-linear (that is, ‘M’ shaped) career pathways may support women GPs to enter (and re-enter) general practice and leadership positions; how part-time work supports work–life balance (or not); and how flexible training pathways may reduce women GPs’ experience of burnout while increasing career opportunities because of the viable alternatives made available for navigating multiple roles (Supplementary Information S7).

With regard to policy, it is strongly recommended that a committee is established, led by both member and non-member organisations (for example, Local Medical Committees, royal colleges, British Medical Association, Medical Women’s Federation), to collectively advocate, and identify ways to collectively advocate, for policy changes specifically relating to changes needed to support women GPs.

Concerning how the research translates to practice, general practice may do well to consider data capture and reporting on metrics relating to gender and thriving across practices. There is also a significant need to locate local practice and area-level initiatives specifically designed to support women GPs, which may not be reported in academic publications or grey literature. There are likely to be novel approaches yet to be explored that move beyond individualised approaches. Box 5 also shows through examples how these can translate and be implemented into practice, although caution is urged against implementing individualised solutions. A guide to informing culture change for women GPs to support this work is also available from the corresponding author.
